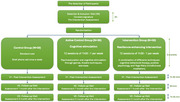# Building resilience for successful aging : developing resources and protective factors to overcome life's challenges with confidence

**DOI:** 10.1002/alz70857_104946

**Published:** 2025-12-25

**Authors:** Marion Ferrandez y Montesinos, Christelle Filleau, Valeria Manera, Xavier Corveleyn

**Affiliations:** ^1^ University of Côte d'Azur, laboratory LAPCOS and laboratory CoBTeK, Nice, Alpes Maritimes, France; ^2^ centre hospitalier universitaire Côte d'Azur, CMRR, Nice, alpes maritimes, France; ^3^ CoBTeK (Cognition‐Behaviour‐Technology) Research Lab, Université Côte d'Αzur, Nice, France; ^4^ Université Cote d'Azur, lab LAPCOS, Nice, France

## Abstract

**Background:**

Resilience, a key factor in successful aging, defined as the ability to adapt positively to adversity. Research shows that resilience protects against cognitive decline and is associated with better cognitive functioning, greater autonomy, improved quality of life, and enhanced psychological well‐being. As a key factor in aging well, fostering resilience in older adults is crucial. However, there is limited evidence on whether resilience can be improved in individuals with neurocognitive disorders. Given its protective benefits, the study aims to evaluate the effectiveness of a mixed intervention to enhance resilience in people over 60 with mild neurocognitive disorders.

Through a 3‐month program, we test a carefully designed intervention combining cognitive‐behavioral therapy, positive psychology, and Yoga Nidra (mindfulness meditation).

**Method:**

The study will involve 90 participants, who will be randomised into three groups: a control group (*N* = 30), an active control group receiving cognitive stimulation (*N* = 30), and an intervention group receiving our resilience‐enhancing program (*N* = 30). The two groups taking part in an intervention (cognitive stimulation and resilience‐enhancing program) will come to the memory clinic for their sessions : one session a week for 1h30 for 3 months. Participants in the control group will receive a brief call once a week for 3 months too. All participants will be assessed at four points: before the intervention, after the intervention, 1 month after the end of the intervention, and finally 3 months after the end of the intervention to see the long‐term effects.

**Result:**

The study is scheduled to start in mid‐February. The participants will finish their three‐month intervention at the end of May and then make their first follow‐up assessment at the end of June. Therefore, we will have most of the results to present to you at the conference.

**Conclusion:**

The results of this study could significantly alter how we approach aging, empowering older adults to not only survive but thrive. This study will provide the keys to designing programs to improve resilience specifically in older adults suffering from mild neurocognitive disorders.